# Coordinating Role of RXRα in Downregulating Hepatic Detoxification during Inflammation Revealed by Fuzzy-Logic Modeling

**DOI:** 10.1371/journal.pcbi.1004431

**Published:** 2016-01-04

**Authors:** Roland Keller, Marcus Klein, Maria Thomas, Andreas Dräger, Ute Metzger, Markus F. Templin, Thomas O. Joos, Wolfgang E. Thasler, Andreas Zell, Ulrich M. Zanger

**Affiliations:** 1 Center for Bioinformatics Tuebingen (ZBIT), University of Tuebingen, Tuebingen, Germany; 2 Dr. Margarete Fischer Bosch-Institute of Clinical Pharmacology, Stuttgart; 3 University of Tuebingen, Tuebingen, Germany; 4 Systems Biology Research Group, University of California, San Diego, La Jolla, California, United States of America; 5 NMI Institute of Natural and Medical Sciences, Reutlingen, Germany; 6 Department of General, Visceral, Transplantation, Vascular and Thoracic Surgery, Hospital of the University of Munich, Munich, Germany; Memorial Sloan-Kettering Cancer Center, UNITED STATES

## Abstract

During various inflammatory processes circulating cytokines including IL-6, IL-1β, and TNFα elicit a broad and clinically relevant impairment of hepatic detoxification that is based on the simultaneous downregulation of many drug metabolizing enzymes and transporter genes. To address the question whether a common mechanism is involved we treated human primary hepatocytes with IL-6, the major mediator of the acute phase response in liver, and characterized acute phase and detoxification responses in quantitative gene expression and (phospho-)proteomics data sets. Selective inhibitors were used to disentangle the roles of JAK/STAT, MAPK, and PI3K signaling pathways. A prior knowledge-based fuzzy logic model comprising signal transduction and gene regulation was established and trained with perturbation-derived gene expression data from five hepatocyte donors. Our model suggests a greater role of MAPK/PI3K compared to JAK/STAT with the orphan nuclear receptor RXRα playing a central role in mediating transcriptional downregulation. Validation experiments revealed a striking similarity of RXRα gene silencing versus IL-6 induced negative gene regulation (r_s_ = 0.79; P<0.0001). These results concur with RXRα functioning as obligatory heterodimerization partner for several nuclear receptors that regulate drug and lipid metabolism.

## Author Summary

During inflammation, circulating proinflammatory cytokines such as TNFα, IL-1ß, and IL-6, which are produced by, e.g., Kupffer cells, macrophages, or tumor cells, play important roles in hepatocellular signaling pathways and in the regulation of cellular homeostasis. In particular, these cytokines are responsible for the acute phase response (APR) but also for a dramatic reduction of drug detoxification capacity due to impaired expression of numerous genes coding for drug metabolic enzymes and transporters. Here we used high-throughput (phospho-)proteomic and gene expression data to investigate the impact of canonical signaling pathways in mediating IL-6-induced downregulation of drug metabolism related genes. We performed chemical inhibition perturbations to show that most of the IL-6 effects on gene expression are mediated through the MAPK and PI3K/AKT pathways. We constructed a prior knowledge network as basis for a fuzzy logic model that was trained with extensive gene expression data to identify critical regulatory nodes. Our results suggest that the nuclear receptor RXRα plays a central role, which was convincingly validated by RXRα gene silencing experiments. This work shows how computational modeling can support identifying decisive regulatory events from large-scale experimental data.

## Introduction

In a variety of acute and chronic illnesses, including bacterial or viral infection, tissue injury, many chronic diseases and most cancers, proinflammatory cytokines such as interleukin (IL) 6, IL-1β, and TNFα evoke a major reorganization of hepatic gene expression resulting in the massive synthesis of acute phase proteins such as C-reactive protein (CRP) [1]. It has long been known that under such conditions the drug metabolism capacity and other hepatic functions can be impaired, largely due to strong and broad downregulation of most drug metabolizing enzymes and transporters (DMET) at the transcriptional level [2–4]. As 60 to 80% of all used drugs are extensively metabolized in the liver [5], these changes may lead to unrecognized drug overdosing and adverse events especially for drugs with narrow therapeutic index, including many cardiovascular, anti-cancer and central nervous system drugs [6–9]. DMET genes are regulated at the constitutive level by hepatic nuclear factors (HNF) such as HNF-1α, HNF-4α, and CCAAT-enhancer binding proteins (C/EBPs) [10,11], while inducible expression involves several ligand-activated receptors including the aryl hydrocarbon receptor (AhR), the constitutive androstane receptor (CAR), the pregnane X receptor (PXR), the peroxisome proliferator-activated receptor-α (PPARα) and others, which function as pleiotropic sensors for a large variety of endogenous and xenobiotic compounds [12,13]. The potential involvement of several of these transcription factors in the downregulation of DMETs by proinflammatory cytokines has been suggested in numerous reports on both mouse and human model systems [14–18]. Taken together, current evidence indicates that the downregulation of hepatic DMET genes by proinflammatory cytokines involves intense crosstalk between signaling components and the transcriptional machinery, potentially involving several and overlapping receptor-dependent mechanisms. Some authors also suggested coordinated mechanisms, e.g. involving the major hepatic retinoid X receptor, RXRα, which is required as a heterodimerization partner for several nuclear receptors including CAR, FXR, LXR, PPAR, PXR, and VDR [19,20].

Further upstream, the signaling pathways involved in DMET regulation also remained largely unclear. IL-6 is known to activate janus kinase/ signal transductors and activators of transcription (JAK/STAT), mitogen activated protein kinase/ extracellular regulated kinase (MAPK/ERK), and phosphoinositide 3 kinase (PI3K)/AKT pathways [21,22]. Earlier work has shown that downregulation of the major human drug metabolizing cytochrome P450, CYP3A4, in response to IL-6 occurs independently of the JAK/STAT pathway [14], although it remained unknown whether this also applies to other DMET genes. On the other hand there is evidence that MAPKs are able to phosphorylate nuclear receptors, which may lead to their subcellular relocalization [19,23], and PI3K/AKT may induce nuclear translocation of NF-κB, which has been shown to antagonize nuclear receptor function by mutual repression as well as by direct binding of NF-κB to DMET promoter regions [24].

To enhance understanding of the complex interactions within signaling pathways and transcriptional networks, different kinds of systems biology modeling techniques have been increasingly employed [25–30]. The most prominent types of logical models are Boolean models, which permit individual components to be only in active or inactive state, thus allowing only a qualitative description of the input-output behavior of signaling pathways. While very large Boolean models can be constructed, they are often not adequate for describing biological reality. By contrast, logic-based ordinary differential equation (ODE) modeling enables a more quantitative simulation of signaling dynamics over time [27]. However, the requirement for extensive time-resolved experimental data as well as prior knowledge about the involved signaling mechanisms limits application of ODE modeling to small networks. An intermediate alternative is provided by “fuzzy logic”, a highly flexible methodology that enables system component states to be in a continuous interval. Recent studies have shown that fuzzy logic can be applied to complex biological problems. Some studies established the use of fuzzy logic to convert prior (e.g., literature-based) knowledge networks to computable models that can be trained to multi-factorial experimental data in order to understand complex signaling pathways [28–30].

Here we used primary human hepatocytes (PHH) stimulated by IL-6, the most potent mediator of the acute phase response in liver, to characterize cellular responses in high-throughput quantitative gene expression and (phospho-)proteomics data sets. Using a previously developed large-scale Boolean model of IL-1 and IL-6 signaling [22] and extensive literature survey we constructed a fuzzy logic model comprising IL-6 signal transduction and DMET gene regulation. Selective inhibitors used in perturbation experiments to disentangle JAK/STAT, MAPK, and PI3K signaling pathways generated the necessary data for model training. Our approach suggests a major role of MAPK and PI3K pathways with the orphan nuclear receptor RXRα at a central position as link between inflammatory signaling and downregulation of drug detoxification genes. We finally validated these findings by RXRα knock-down experiments. This study emphasizes fuzzy logic modeling as a useful alternative to elucidate complex signaling interactions.

## Results

### Construction of a prior knowledge network

We first constructed a prior knowledge network (PKN) comprising IL-6 signal transduction and downstream gene regulation (S1 Fig). The core of the signal transduction part of the network was taken from the Boolean model by Ryll and colleagues [22], which comprises several signaling pathways. As we were primarily interested in identifying logical nodes and not in dynamic features, we did not attempt to include a time scale. This allowed us to simplify the model by removing feedback loops, including those involving SOCS1 and SOCS3, without negatively impacting our model, because feedback loops by definition require a time scale. Furthermore, we deleted several input and output nodes not relevant for our study. The AND, OR, and NOT gates of the remaining network were transformed into activating or inhibiting transitions from the respective input species of the gate to the output species. All changes introduced to the model by Ryll et al. are summarized in S1 Text.

The IL-6 signal transduction module of the PKN was supplemented with a gene regulation module by compiling biological knowledge from various cell types as provided by databases and scientific literature, including e.g., BIOBASE TRANSFAC and Pubmed (S2 Text). The resulting network contained all DMET genes including their transcriptional regulators STAT3, NF-κB, AhR, HNF-1α, HNF-4α, ELK1, glucocorticoid receptor, and cFOS, as well as a species RXR/NR, which represents the complexes between RXRα and any of the nuclear receptors known to be a potential partner of RXRα.

### Phosphoproteomics analysis of IL-6 response pathways in PHH

**Fig 1:**
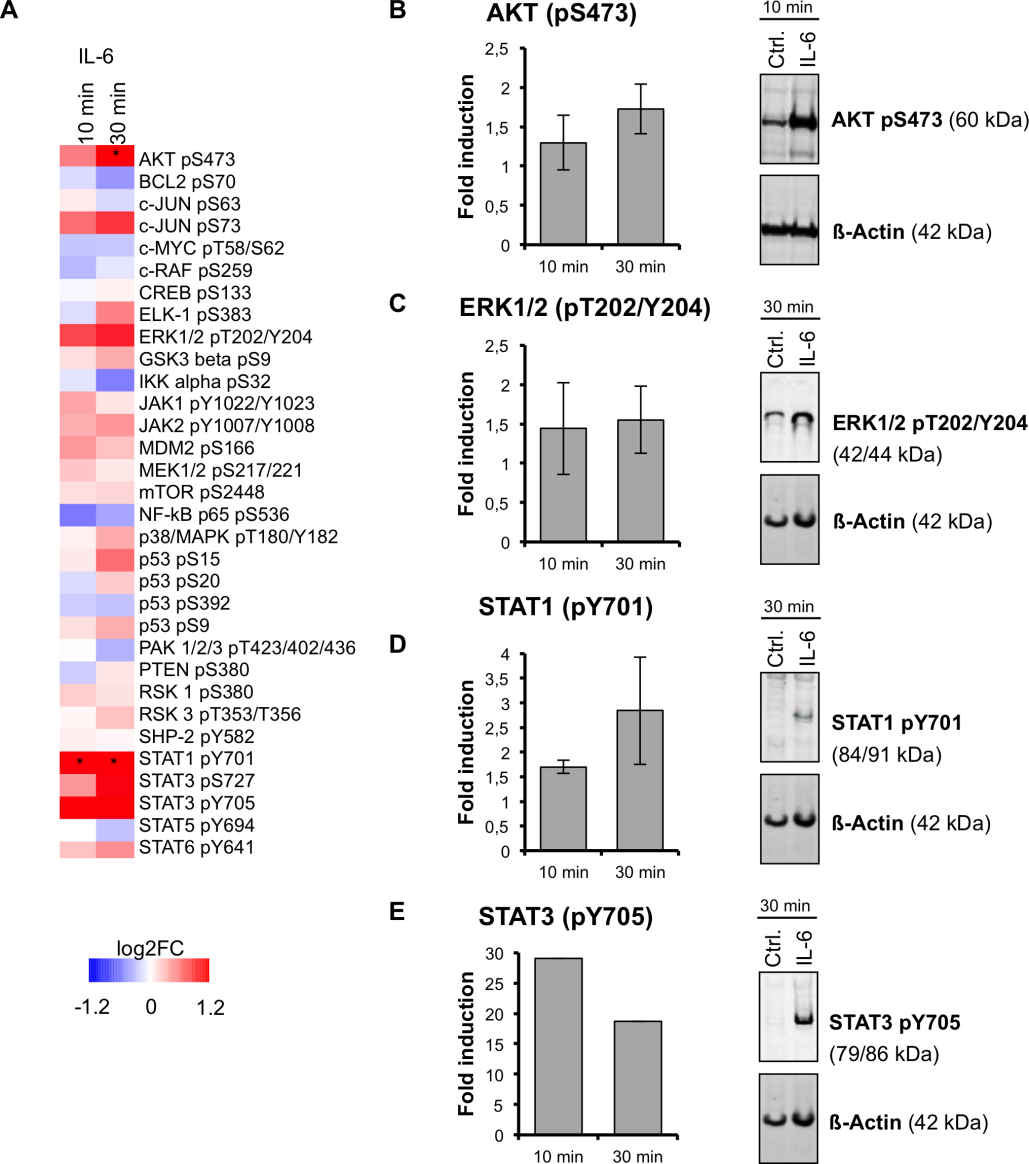
Phosphoprotein activation upon IL-6 stimulation in PHH. **(A) Heat map showing mean changes (IL-6 vs. control) in levels of 32 phosphoproteins at two time points from three independent PHH donors.** Phosphoproteins were detected by RPA with phosphospecific antibodies directed at the indicated phosphorylation sites. Red color represents positive and blue color negative log2FC (color code provided). Asterisks indicate statistical significance (P<0.05). (B-E, left panel) Bar charts showing means ± SD (n = 3) of relative fluorescent intensities (RFIs) of selected phosphoproteins at different time points after IL-6 stimulation. RFIs were obtained from the RPA and background-normalized. Error bars represent standard deviations calculated from replicates of three donors (not available in E) and asterisks indicate statistical significance (P<0.05). (B-E, right panel) Western blots of total cell lysate (20 µg each) of selected phosphoproteins from control (Ctrl.) and IL-6 treated cells. β-Actin staining served as loading control. Detection was performed with an Odyssey infrared imaging system.

We used PHHs because these cells are considered the “gold standard” model for the investigation of hepatic metabolism of drugs and its regulation at the cellular level [31,32]. Despite considerable inter-individual variability and limitations in availability, PHHs are superior to immortalized cell lines, whose de-regulated cell cycle control is a result of massive changes in mitogenic and apoptotic signaling, and to primary mouse hepatocytes, whose genomic response poorly resembles that of humans, especially during an inflammatory response [33]. It should be pointed out that the limited availability of PHH, their rather short live-span in the fully differentiated state, and the lack of appropriate cryopreservation protocols preclude the possibility to perform complete experiments or replications in the same donor. We determined signaling pathway activation upon IL-6 stimulation in PHH of three independent donors (donors D1-D3, Table 1) by quantification of a large panel of phosphoproteins using reverse-phase protein array (RPA) technology (Fig 1A). Among the 32 detected phosphoproteins, consistently induced phosphorylations (i.e. at 10 and 30 min after stimulation) of AKT, c-JUN, ERK1/2, STAT1, STAT3, and STAT6 were observed, although not all were statistically significant (Fig 1B–1E, left panel). Western blot analyses confirmed the RPA findings (Fig 1B–1E, right panels). Thus, increased phosphorylation of AKT, ERK1/2, STAT1, and STAT3 at their respective phosphorylation sites was demonstrated, indicating activation of the associated signaling pathways.

**Table 1:** Overview on hepatocyte donors, treatments, and analyses.

Donor #	Age	Sex	Treatments	Analyses	Utilisation
D1	75	f	IL-6	phosphoproteins (10 + 30 min)	signaling pathways
D2	71	f	IL-6	phosphoproteins (10 + 30 min)	signaling pathways
D3	48	f	IL-6	phosphoproteins (10 + 30 min)	signaling pathways
D4	59	f	IL-6, IL-6 + PI3Ki	gene expression (24 h)	modeling
D5	47	f	IL-6, IL-6 + MAPKi + PI3Ki	gene expression (24 h)	modeling
D6	29	f	IL-6, IL-6 + STAT3i	gene expression (24 h)	modeling
D7	48	m	IL-6, IL-6 + STAT3i + MAPKi	gene expression (24 h)	modeling
D8	71–80	f	IL-6, IL-6 + MAPKi, IL-6 + PI3Ki, IL-6 + STAT3i	gene expression (24 h)	modeling
D9	21–30	m	IL-6, siRXRα	gene expression (24 h)	validation
D10	41–50	f	IL-6, siRXRα	gene expression (24 h)	validation
D11	70–79	m	IL-6, siRXRα	gene expression (24 h)	validation

### Chemical perturbation analysis of IL-6 response pathways in PHH

**Fig 2:**
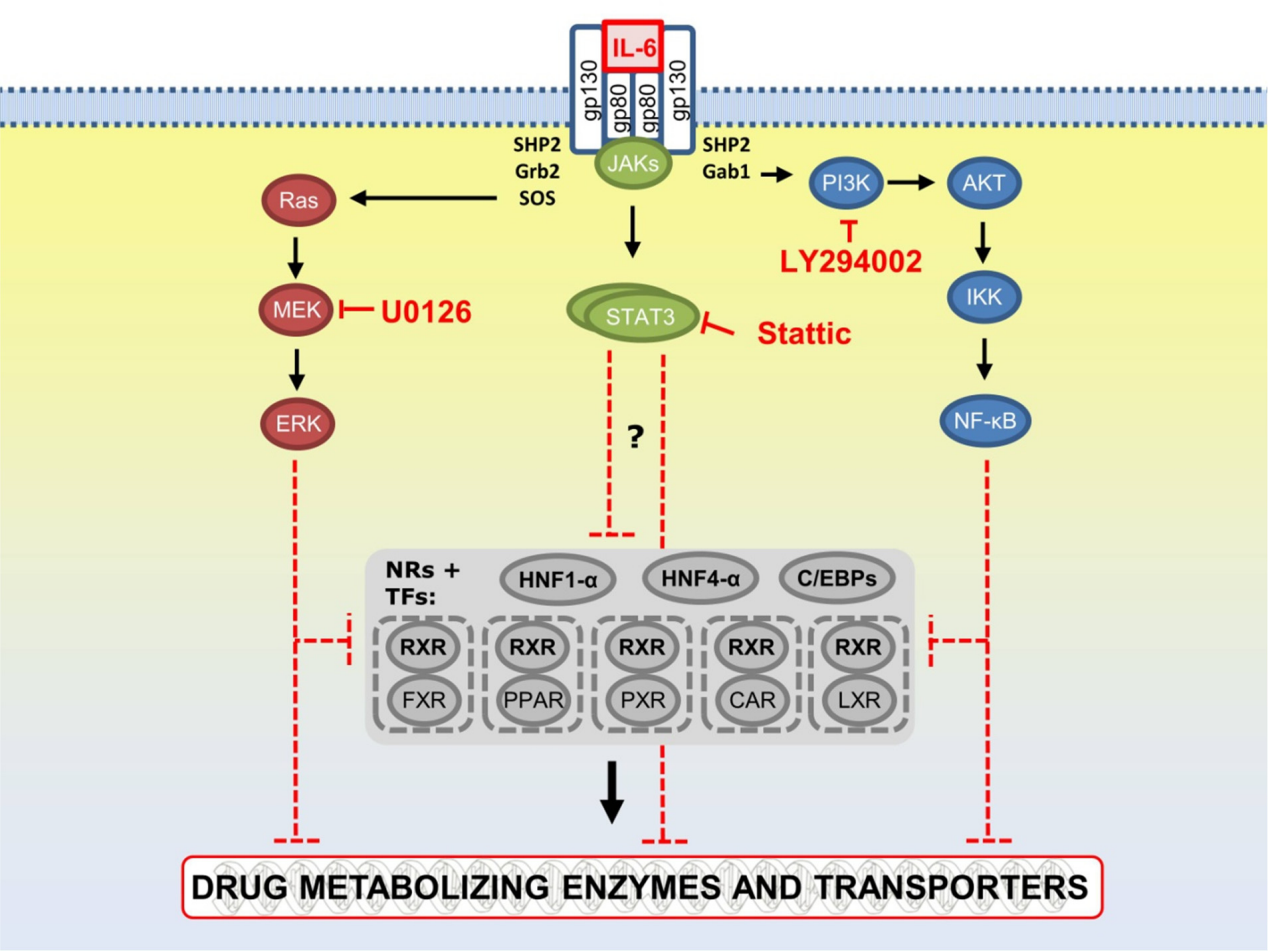
Overview of IL-6-induced pathways and their inhibition by selective inhibitors. Classically, IL-6 signals through a receptor complex composed of glycoprotein 130 (gp130) and gp80 (IL-6Rα), or the soluble sIL-6R and activates the Janus kinase (JAK)/signal transducer and activator of transcription (STAT) pathway. Apart from STAT transcription factors, IL-6 also activates the mitogen activated protein kinase (MAPK)/extracellular regulated kinase 1 and 2 (ERK1/2) (MAPK/ERK)-cascade and the phosphatidyl-inositol-3-kinase (PI3K)-cascade, both through the proximal SHP2 binding phosphotyrosine motif, leading to activation of AKT serine/threonine kinases. AKT may then transiently associate with and induce the activation of IκB kinase and thereby activate the canonical NF-κB pathway. Colors indicate major pathways: PI3K/AKT (blue), JAK/STAT (green), and MAPK (red). The grey box summarizes nuclear receptors (NRs) and transcription factors responsible for the inducible or constitutive regulation of DMET genes. Arrows indicate positive stimulation or transcriptional activation, whereas flat-ended arrows indicate inhibition, mutual repression or transcriptional repression. Points of action of chemical pathway inhibition by LY294002 (PI3K inhibition), Stattic (STAT3 inhibition), and U0126 (MAPK inhibition) are shown by red flat-ended arrows. This figure was adapted and modified from Eulenfeld et al. [21] and Castellano and Downward [55] using additional primary literature sources (S2 Text). For the complete PKN please refer to S1 Fig.

Gene expression upon IL-6 stimulation was measured after 24 h for major DMET genes as well as for genes indicating inflammation or activation of a specific pathway (see S1 Table for a list of all measured genes). We used specific chemical inhibitors to selectively interfere with STAT3, PI3K, and MAPK signaling as confirmed by RPA measurements (Fig 2). Gene expression analysis following chemical inhibitions of pathways were conducted in PHH from five liver donors (donors D4-D8, Table 1). The resulting five gene expression data sets contained single inhibitions of STAT3, PI3K, and MAPK as well as combinatorial inhibitions of STAT3 + MAPK and PI3K + MAPK, while the combined inhibition of STAT3 + PI3K rapidly induced cell death and was therefore not included in the analysis. In agreement with previous observations, IL-6 elicited a profound transcriptional downregulation of many genes of the drug detoxification system.

**Fig 3:**
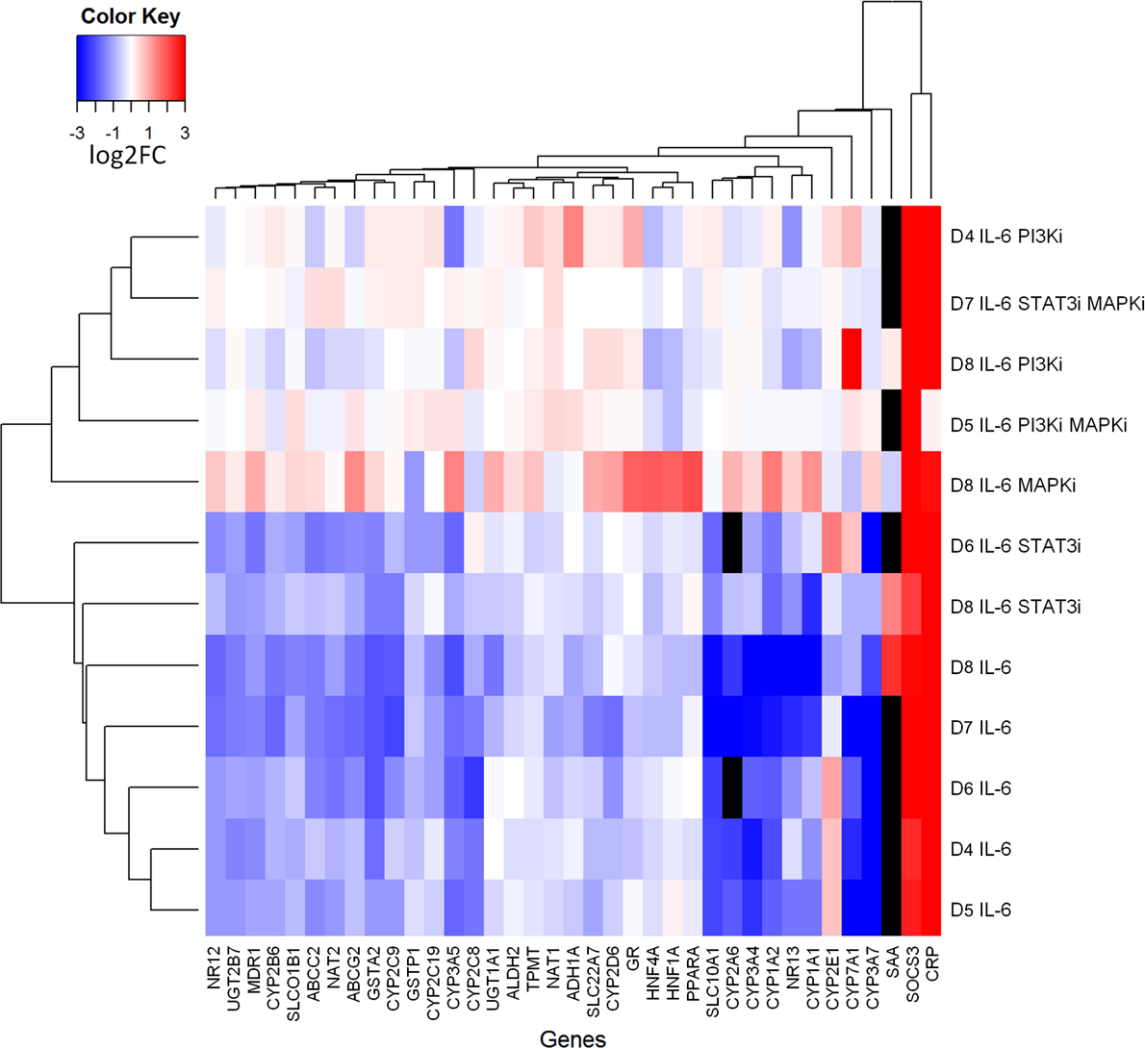
Heat map of gene expression changes following IL-6 stimulation and chemical inhibitions in PHHs of five donors (D4-D8; Table 1). Cells were treated for 24h with IL-6 in the absence or presence of one or two chemical inhibitors (PI3Ki: LY294002; MAPKi: U0126; STAT3i: Stattic; see Table 1 and Materials and Methods for details). Gene expression was quantified by TaqMan qPCR and fold changes relative to control (vehicle alone) are shown for 37 genes contained in the model; missing measurements are displayed in black color. See Materials and Methods for details on heat map construction and hierarchical clustering.

Hierarchical clustering based on the log 2 linear fold change (log2FC) values shows major clusters of genes and treatments (Fig 3). One major gene cluster comprises *CRP* and *SOCS3*, which were strongly upregulated, indicating IL-6-dependent activation of the acute phase response. Except for *CYP2E1*, all *CYP*s as well as several important ATP-binding cassette (*ABC*) and solute carrier (*SLC*) transporters were downregulated upon IL-6 stimulation. The upregulation of *CYP2E1* was not seen in all donors, causing a separation from the two other upregulated genes in the hierarchical clustering. The cluster of treatments shown on the upper part of Fig 3 consisted of treatments including a MAPK and/or a PI3K inhibitor. Most of the IL-6-induced effects were attenuated in this cluster. Single treatments with the STAT3 inhibitor clustered together with the five IL-6 treatments in the absence of inhibitors, although almost complete loss of STAT3 pY705 was demonstrated by RPA analysis (see Materials and Methods). In conclusion, both MAPK and PI3K signaling appeared to play a more important role in IL-6 induced DMET gene regulation as compared to STAT3.

### Construction and application of an optimized fuzzy logic model

**Fig 4:**
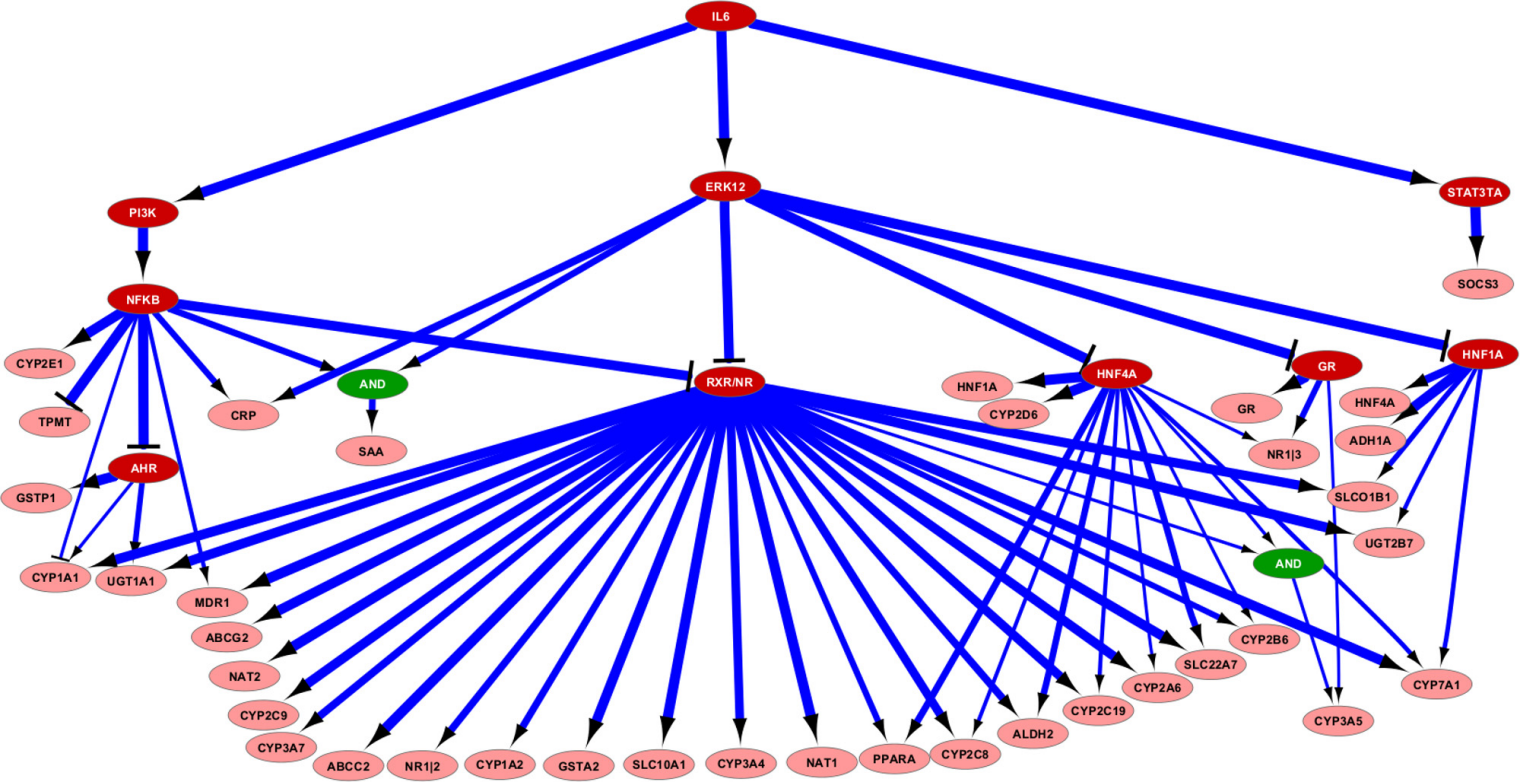
Optimized model family based on the PKN and the gene expression perturbation data. Signaling molecules are displayed in white text on red background, genes in black text on light red, and AND-nodes in green ovals. Only transitions that are contained in at least 30% of the 100 optimized models are shown. The line width of each transition represents the percentage of the 100 optimization runs, in which the transition is retained in the model. RXR/NR denotes heterodimeric complexes between RXRα and various nuclear receptors which maintain transitions to most of the DMET genes. The figure has been created with Cytoscape [56].

We used adapted routines of the R library CNORfuzzy [34] in order to create an optimized fuzzy logic model from the gene expression data and the prior knowledge network (see Materials and Methods). The model was trained with the five perturbed gene expression data sets that contained single inhibitions of STAT3, PI3K, and MAPK as well as combinatorial inhibitions of two pathways (donors D4-D8, Table 1). The resulting model family (Fig 4) illustrates the connections between signaling molecules and regulated genes quantitatively, with the line width of each transition representing the percentage of the optimized models (N = 100) containing this particular transition. By far most of the transitions are connected to the RXR/NR complex species, which represents heterodimeric complexes between RXRα and a number of nuclear receptors including PXR, FXR, and others [20]. Additional striking nodes that connect to several regulated genes are identified as NFkB, HNF4A, and HNF1A. For a few genes the model further suggests the inhibition of the glucocorticoid receptor by MAPK, and inhibition of AHR by NF-κB. The involved transitions for these events are present in nearly all of the optimized models (Fig 4).

**Fig 5:**
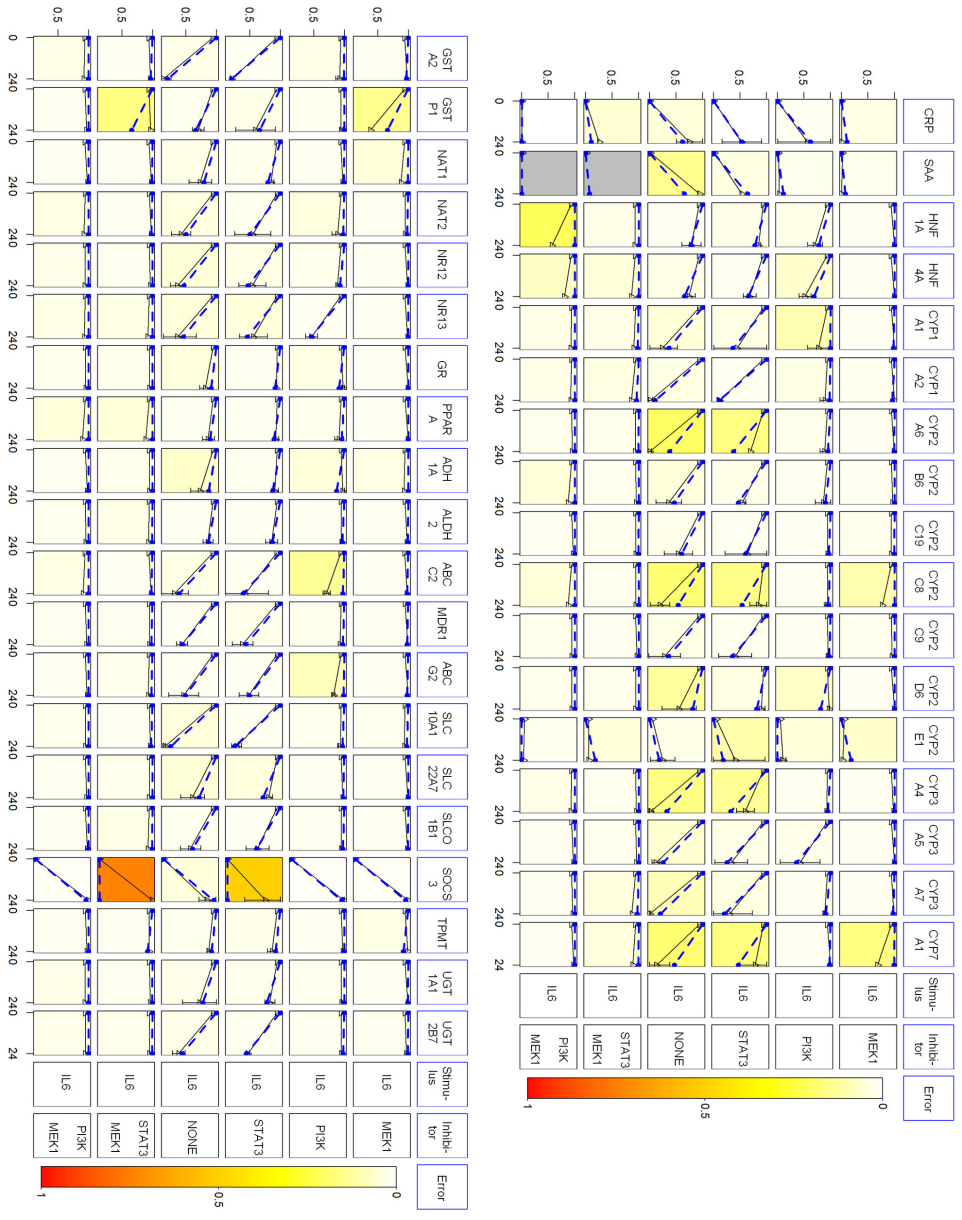
Comparison of the predictions of the optimized PKN with the respective data points from five gene expression data sets. The normalized data points are displayed in black solid lines, the predicted points in blue dashed lines. The vertical bars show the standard deviations of the normalized data points. The columns correspond to the genes, the rows to the different treatments. The figure has been created with an adapted method of CNORfuzzy [34].

Comparison of the predictions of the model family with the respective data showed marked agreement for most genes, as indicated by bright-colored fields (Fig 5). Some higher deviations between prediction and data were seen only for few genes in certain conditions, in particular SOCS3 and to a lesser extent some DMET genes, e.g. CYP2A6 and CYP2C8, in the presence of STAT3 inhibitor (darker colored fields). This may indicate unknown regulatory events not contained in the prior-knowledge network. In the case of SOCS3, the deviations reflect induced measured levels while the model predicts baseline levels due to the assumed inhibition of STAT3. Despite confirmed effective STAT3 inhibition (*vide supra*), we cannot exclude the possibility that residual activity leads to “leaky” upregulation of SOCS3, since this is one of the most strongly regulated STAT3 target genes. It should be pointed out that this discrepancy cannot be due to the missing feedback loop in our model, which precludes secondary effects, as mentioned above.

### Validating the role of RXRα

**Fig 6:**
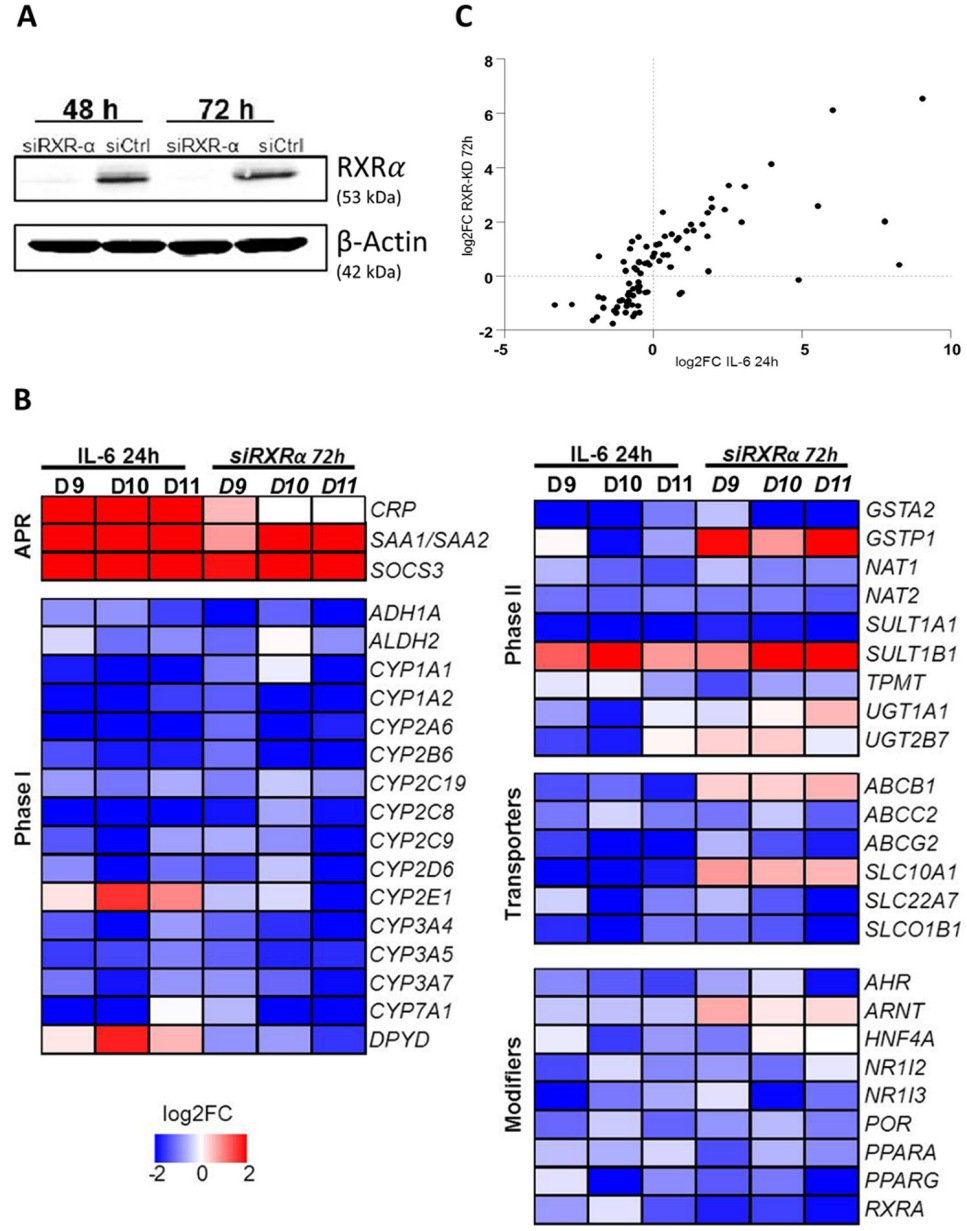
Validation of the role of RXRα in the downregulation of DMET genes by gene silencing in PHH. (A) Western blot analysis of RXRα in total protein lysates 48 h and 72 h after transfection of cells with siCtrl (control) and siRXRα. β-Actin staining served as loading control. (B) Heat map showing the relative changes in gene expression (log2FC) for 86 selected APR and DMET genes after IL-6 stimulation (IL-6 vs. control, 24 h) or after siRNA-mediated KD of RXRα (siRXRα vs. siControl, 72 h) in PHH of three independent donors (D9-D11, Table 1). Red color represents up- and blue color downregulation according to the supplied color code. Mean fold changes and statistical analyses of the same data are presented in S3 Fig. (C) Spearman correlation analysis between mean log2FC values for IL-6 and siRXRα treatments across all genes for all three donors. The calculated Spearman coefficient was r_s_ = 0.79 (N = 86, P<0.0001).

The optimized fuzzy logic model (Fig 4) suggested an important role of the RXRα/NR complexes. We used siRNA-mediated selective RXRα gene silencing to analyze the impact on gene expression of major DMET genes via high-throughput real-time qPCR analysis. RXRα protein expression was almost completely suppressed as demonstrated by Western blot analysis (Fig 6A). Fig 6B illustrates the IL-6- and RXRα knock-down (KD)-induced gene expression changes in PHH from three independent donors (D9-D11, Table 1). RXRα KD was further confirmed by more than 90% downregulation of *RXRA* mRNA. Upon IL-6 stimulation, APR genes were highly upregulated and a coordinated downregulation of major DMET genes was observed in all donors, similar to those described for donors D4-D8 (Fig 3). The impact of RXRα KD on expression of DMET and modifier genes was very pronounced with similar patterns compared to the effects of IL-6. This visual impression was supported by Spearman correlation analysis, showing a highly significant correlation between the mean fold changes of IL-6 and RXRα KD treatments (r_s_ = 0.79; N = 86; P<0.0001; Fig 6C). Among the phase I metabolism genes, most of which were strongly and significantly downregulated, only *CYP2E1* reacted differently, being downregulated by the RXRα KD while it showed (nonsignificantly) higher levels after IL-6 treatment. The phase II metabolism genes *NAT1*, *NAT2*, *and SULT1A1* were also downregulated to similar extent. Of note, the transporters *ABCB1* and *SLC10A1* showed opposite regulation, being upregulated by RXRα KD and downregulated by IL-6. Among the DMET modifiers, only *AHR*, *ARNT*, and *PPARA* expression was significantly impaired by IL-6 but not after the KD of RXRα. As expected, the RXRα KD experiment did not indicate significant induction of most acute phase genes.

## Discussion

In this study we investigated the response of primary human hepatocytes to stimulation with IL-6, the most potent pro-inflammatory cytokine for the hepatic APR. Using quantitative gene expression and time-resolved (phospho-)proteomics data sets of unprecedented comprehensiveness, we optimized a fuzzy logic model comprising all known major IL-6 signal transduction pathways as well as a broad spectrum of DMET gene regulation pathways. Our model suggested a major role of MAPK and PI3K pathways with the orphan nuclear receptor RXRα playing a central role as link between inflammatory signaling and downregulation of drug detoxification genes. Experimental RXRα knock-down by RNA interference further substantiated a coordinating role of RXRα to downregulate a wide variety of drug detoxification genes during inflammation.

Based on our high-throughput (phospho-)proteomic analysis only a few major signaling molecules demonstrated increased phosphorylation status following IL-6 treatment: AKT (S473), ERK1/2, (T202/Y204), STAT1, (Y701) and STAT3 (Y705). Whereas activation of STATs as well as established APR factors and ERK1/2 by IL-6 was shown previously [21,35,36], increased phosphorylation of AKT at S473 following IL-6 treatment has not been shown before to our knowledge. Of note, PHH cultures of several donors showed increased phosphorylation at AKTS473 already at the steady state prior to treatment. This may indicated drug- or disease-induced basal deregulation of PI3K signaling pathway in the hepatocytes of these patients [37]. Our chemical inhibitions, which had been confirmed to be effective at the used concentrations by RPA phosphorylation analysis, indicated that blocking STAT3 signaling pathway compromised the IL-6 effect on DMET mRNA expression only marginally and only for few DMET genes, in agreement with a previous study showing that STAT3 was not required for CYP3A4 downregulation [14]. Inhibition of the MAPK and PI3K pathways however markedly interfered with IL-6-induced DMET expression changes, particularly in combination (Fig 3). Thus, co-inhibition analyses of PI3K and MAPK as well as of STAT3 and MAPK signaling pathways abolished almost all IL-6-mediated effects on DMET gene expression, suggesting a higher relevance of MAPK/PI3K compared to the JAK/STAT pathway in mediating the IL-6 triggered effects. Some limitations of this approach should be noted. Chemical inhibitors may have unspecific effects [38] and may also activate NRs by themselves [39]. However, we believe that the concentrations used here were low enough to show primarily true effects on the intended pathways. Furthermore, extensive pathway crosstalk [21] poses general difficulties in the interpretation of such data.

In order to elucidate the regulatory events responsible for IL-6 regulation of DMET gene expression, we developed a fuzzy logic model. This modeling technique avoids the requirement for estimating numerous kinetic parameters as in ODE modeling and allows more realistic approximations to biological systems compared to simple Boolean logic. Fuzzy logic modeling has been previously used in studies involving rather tedious manual calibration of model parameters [28] as well as parameter estimations with heuristic optimization routines [29,30]. In our study we applied the latter approach due to the lack of prior knowledge about model parameters. This required training of the model by experimental data. Morris et al. [29] introduced a method to train signal transduction pathways to protein data that we adapted to the use of gene expression data sets. As we combined several data sets for model training, we constructed a “mean” model over these data sets. A principal problem in this respect could be variability in the gene expression data throughout different donors. However, as shown by our datasets representing 8 individual donors (Figs 3 and 6), IL-6 effects on both APR and DMET genes were remarkably similar for all liver donors. In principle, donor-specific models could have been created by model calibration with respect to only the data for a specific donor. In order to obtain a reliable model, this would, however, require that all experimental perturbations are conducted in this donor, which is practically very difficult with PHHs.

The model suggested the inhibition of the complexes of RXRα and nuclear receptors by MAPK and Nf-κB as the major event for the downregulation of most DMET genes by IL-6. RXRα is required as heterodimerization partner for several important nuclear receptors including CAR, FXR, LXR, PPAR, PXR, and VDR [20]. A coordinating role of RXRα based on its biological function has been previously proposed [19] but only few mouse genes had been observed in that study and to our knowledge the hypothesis has not been tested for humans. Here we used siRNA-mediated gene KD in PHH to confirm the model-proposed role of RXRα in DMET gene downregulation. In three independent donors we observed highly similar patterns of regulation with comparably few interindividual differences (Fig 6), resulting in a highly significant correlation (r_s_ = 0.79, N = 86; P<0.0001) between mean fold changes elicited by the two treatments.

Regarding the underlying mechanisms, it had been shown previously that endotoxin leads to rapid loss of nuclearly localized RXRα, while RXRα mRNA levels were not affected [19], which is in agreement with our findings (Figs 6 and S3). The detailed molecular events leading to RXRα inhibition remain to be investigated. Modulation of the phosphorylation status of nuclear receptors including RXRα has been proposed as a possible event in this process [19].

In conclusion, this study provides new insights into the coordinated negative regulation of DMET genes by the proinflammatory cytokine IL-6. Using extensive datasets that characterize the activation of signaling pathways and the regulation of a broad range of APR and DMET genes in primary human hepatocytes we found that MAPK and PI3K/AKT signaling pathways appear to be more important than STAT3 signaling in mediating the response of DMET genes to IL-6. A fuzzy logic model based on gene expression data sets from five different hepatocyte donors identified RXRα as a key player in downregulation of DMET gene expression, which was confirmed by gene silencing experiments. While previous fuzzy logic modeling approaches mainly focused on describing signaling events, our model also involves gene regulation. Hence, our study is a novel example for the elucidation of key gene-regulatory events from biological data and prior knowledge using fuzzy logic.

## Materials and Methods

### Ethics statement

The use of PHH was approved by the local ethics committee and written informed consent was obtained from all donors (number 025–12, Ethics Committee of the Medical Faculty of the Ludwig-Maximilians-Universität München).

### Reagents

William’s E Medium was purchased from Invitrogen Life Technologies (Darmstadt, Germany). Fetal bovine serum (FBS) was from PAA Laboratories GmbH (Pasching, Austria), human insulin from Sanofi (Frankfurt, Germany), and hydrocortisone from Pfizer Pharma GmbH (Karlsruhe, Germany). Hepes, L-glutamine, MEM non-essential amino acids (NEAA), penicillin/streptomycin (Pen/Strep), phosphate-buffered saline (PBS), and sodium pyruvate were purchased from GIBCO (Carlsbad, CA, USA). Bovine serum albumin (BSA), dexamethasone, and dimethyl sulfoxide (DMSO) were from Sigma-Aldrich (Steinheim, Germany), hydrocortisone from Pfizer (Karlsruhe, Germany). Human recombinant intereukin-6 (IL-6) was purchased from Promo Cell GmbH (Heidelberg, Germany). Human recombinant interleukin 1β (IL-1β) and tumor necrosis factor α (TNF α) were purchased from Sigma-Aldrich (Steinheim, Germany). All cytokines were reconstituted and stored as high concentration stocks according to manufacturer specifications. Chemical inhibitors were purchased from the following suppliers: LY294002 (Merck, Darmstadt, Germany), U0126 (Promega, Madison, WI, USA), and Stattic (Sigma-Aldrich, Steinheim, Germany). Inhibitor stock solutions (20 mM each) were prepared in DMSO. All TaqMan assays were purchased from Applied Biosystems (Foster City, CA, USA). Silencer Select Pre-designed siRNA was purchased from Applied Biosystems (Foster City, CA, USA).

### Human hepatocyte cultures

PHH were isolated from partial liver resections by collagenase digestion as described previously [32,40]. Donor data are shown in Table 1. Isolated cells with a viability of more than 70% as determined via trypan exclusion test were seeded at a density of 4 × 10^5^ viable cells/well onto BioCoat Collagen I Cellware 12-well culture plates (Becton Dickinson, Bedford, USA) in William’s E Medium, supplemented with 10% FBS, 100 U/ml Pen/Strep, 2 mM L-glutamine, 32 mU/ml human insulin, 1 mM sodium pyruvate, 1X NEAA, 15 mM hepes, and 0.8 μg/ml hydrocortisone. After 24 h, cells were equilibrated for another 24 h in cultivation medium, containing William’s E Medium, supplemented with 10% FBS, 100 U/ml Pen/Strep, 2 mM L-glutamine, 32 mU/ml human insulin, 0.1% DMSO, and 0.1 μM dexamethasone. Cells were maintained at 37°C in 5% CO2 throughout the experiment with the exception of the shipping period. All cells were cultured for a minimum of 48 h between isolation and treatment. Media were changed every 24 h.

### Treatments

PHH were treated for up to 24 h with 10 ng/ml human recombinant IL-6 in PBS, supplemented with 0.1% BSA, or vehicle only (PBS + 0.1% BSA). This concentration had been previously shown in various cell models to activate STAT3 and to induce CRP expression without being toxic [41,42]. Furthermore, maximal induction of acute phase protein mRNA expression including CRP and SAA1/2 was recently demonstrated by dose-response experiments in PHH [4].

For inhibition of signaling pathways, three specific chemical inhibitors were applied, targeting three major signaling proteins: LY294002 for PI3K (upstream of AKT), U0126 for MEK1/2 (upstream of ERK1/2), and Stattic for STAT3. LY294002 was shown to be a potent inhibitor of PI3K in hepatocytes, where concentrations of > 20 μM inhibited the enzyme’s activity by more than 90% [43]. U0126 is a selective inhibitor for MEK-1 and -2 [44]. It was shown to effectively inhibit wild-type MEK1 phosphorylation of ERK2 in concentrations between 20 and 100 μM in in vitro experiments [45]. Stattic is a selective inhibitor of the activation, dimerization, and nuclear translocation of STAT3 shown to inhibit STAT3 in vitro with an IC_50_ value after one hour of incubation of 5.1 ± 0.8 μM [46]. For inhibition, medium was aspirated and replaced by fresh medium containing one or a combination of chemical inhibitors in final concentrations of 10 μM (Stattic) and 50 μM (LY294002 and U0126). DMSO-treated cells served as control. After incubation for 1 h, cells were treated with IL-6 or vehicle as described above. Successful inhibition of signal propagation was assessed in PHH using phosphoproteomics RPA technology. IL-6-dependent AKT S473, ERK1/2, and STAT3 Y705 phosphorylation was confirmed to be nearly abolished by LY294002, U0126, and Stattic, respectively.

KD of RXRα via Silencer Select Pre-designed siRNA (P/N4392420, #s12384; sense: UCGUCCUCUUUAACCCUGAtt, antisense: UCAGGGUUAAAGAGGACGAtg) was carried out in PHH according to the manufacturer’s instructions. In short, transfection mix was prepared and after 20 min incubation at RT added, giving a total volume of 1.2 ml per well (12-well plate).

### Quantitative real-time PCR

Total RNA was isolated from PHH and HepaRG cells using the RNeasy Mini Kit, including on-column genomic DNA digestion with RNase free DNase Set (Qiagen, Hilden, Germany). The RNA integrity (RIN) and quantity were analyzed with the Agilent 2100 Bioanalyzer using the RNA 6000 Nano Kit (Agilent Technologies, Waldbronn, Germany). Only samples with a RIN value larger than 7 were used. Synthesis of cDNA was performed with 500 ng RNA using TaqMan Reverse Transcription Reagents (Applera GmbH, Darmstadt, Germany). Quantification of expression of 95 genes was performed using Fluidigm’s BioMark HD high-throughput quantitative 96x96 chip platform (Fluidigm Corporation, San Francisco, CA, USA), following the manufacturer’s instructions [47]. All used predesigned TaqMan assays are listed in S1 Table. The mRNA expression levels were normalized to the most stably expressed gene among a selection of housekeeping genes (*ACTB*, *GAPDH*, *GUSB*, *HMBS*, *POLR2A*, *RPLP0*, *TBP*) by using the Normfinder Excel Add-in as described by Andersen and colleagues [48]. Relative gene expression changes were calculated using the delta delta Ct (ΔΔCt) method [49]. ΔΔCt values were calculated by subtracting the ΔCt value of the calibrator sample (e.g., PBS, 0.1% BSA-treated) from the ΔCt of the experimental sample (e.g., IL-6-treated). As the Ct is on a log 2 scale, linear fold changes (FCs) were calculated as 2^(-ΔΔCt)^.

### Quantification of phosphoproteins

RPA technology and Western blot analysis were used for relative quantification of protein phosphorylations. In the RPA, pL amounts of protein mixtures are immobilized in a microarray format and the presence of specific target proteins is screened by using highly selective antibodies [50]. This technology allows for the simultaneous quantification of more than 100 proteins and phosphoproteins by direct two-step immunoassay using specific primary antibodies [51]. Proteins were isolated using the CLB1 lysis buffer. Sample preparation and measurements were carried out as described elsewhere [51]. Western blots of selected phosphoproteins and of RXRα were performed with total cell lysate (20 μg of protein). β-Actin staining served as loading control. Detection was performed with an Odyssey infrared imaging system. Details on the antibodies used can be found in S2 Table.

### Clustering of gene expression data

Each DMET gene is represented by a vector of fold changes for all treatments. The R function heatmap.2 [52] was used to create a heat map of the genes and treatments based on the logarithmized fold changes. The genes as well as the treatments are thereby clustered hierarchically with average-linkage clustering using Euclidean metrics [53].

### Fuzzy logic modeling and CNORfuzzy method

CNORfuzzy is an add-on to the CellNOptR, which constructs a fuzzy logic model that enables the model species to be in a continuous state in the interval [0,1]. A state of 0 for a model species then represents inactivity of the species and a state of 1 the highest possible activity. States in between stand for intermediate activity levels of the species.

This routine has been used with proteomic data [29] and was here applied to gene expression data. The two discrete states for model species in CellNetOptimizer (CNO) corresponding to an active and inactive species have proven to be suitable for modeling the activity of signaling molecules [54]. However, for most genes instead of this on-off-pattern, we rather expect a gene to have several activation states. Therefore, we use CNORfuzzy for creating our model of IL-6 induced DMET gene regulation. CNORfuzzy first tries to remove from the network all species that are neither measured nor perturbed in the experimental data, i.e., the only species that are additionally maintained in the network are those that are necessary for logical consistency. The program then expands the PKN with possible AND-gates to supplement the already implemented OR-gates. For model inference a genetic algorithm that optimizes the mean squared error between model prediction and normalized experimental data was used. This algorithm fits transfer functions for each gate to the data. In the following model reduction step, gates that do not significantly affect the mean squared error (MSE) between model prediction and data are removed based on a chosen selection threshold that determines the maximum tolerated increase in the MSE, when a model is reduced by removing logic gates. CNORfuzzy thus reduces the network to a topology that is sufficient to explain the experimental data.

The genetic algorithm for optimization and the following reduction procedure of CNORfuzzy were run 100 times, resulting in a family of optimized and reduced models. The mean number of parameters in the optimized model family depends on the chosen selection threshold (S2 Fig). At a selected threshold of 0.01, the average MSE for the 100 models was 0.013 and the mean number of parameters in the optimized model family was approximately 110. Details on network compression and optimization are presented in the supplemental S3 Text.

### Adaptation of CNO normalization method for gene expression data

The methods of Cell Net Optimizer and CNORfuzzy are based on normalized data in the interval [0,1]. However, our gene expression data have a different structure and the normalization method is neither suitable for the Ct values, nor for the calculated fold changes. Therefore, we adapt the given method in order to enable a transformation of the fold change values into the interval [0,1].

We transformed all fold change values *fc*_*i*_ of a gene with the following Hill function, which depends on the Hill coefficient *h* and the value *m* standing for the midpoint of the normalization function:

$$v_{i}\;=\frac{{{fc}_{i}}^{h}}{m^{h}+{{fc}_{i}}^{h}}$$

This Hill function is similar to the Hill function in the CNO routine [54]. The main difference to the normalization method provided by CNO is the lack of fold change computation in our approach, because our data already contained fold changes.

The fold change values for genes downregulated by IL-6 are usually smaller than 1 in the experimental data, whereas for genes upregulated by IL-6 they are greater than 1. In order to reasonably transform the values into [0,1] for both classes of genes, the midpoint of the Hill function *m* had to be chosen differently. An important aspect to consider for this transformation is that model simulation with CNORfuzzy only produces species states of 0 or 1 in the case of inactivity of IL-6 (see S3 Text). Therefore, normalization of gene states for control treatments should also lead to values near 0 or 1. For all genes downregulated by IL-6, their gene activity after control treatment must be high compared to after IL-6 treatment and thus their normalized values should be close to 1. To this end, *m* was set to 0.5, which proved effective. For CYP2E1, which is upregulated, we correspondingly set *m* = 2, because the control treatments represent a comparably low activity in this case. The other genes upregulated by IL-6 (SAA, CRP, SOCS3) showed large fold changes upon IL-6 stimulation and *m =* 2 was not suitable for their midpoint of the normalization function. Therefore, we set *m* to half of the mean fold change value of the IL-6 treatments over the data sets.

For all genes *h* was set to 4, which led to a transformation of the control fold change values (1) to a value near 0 (for the genes upregulated by IL-6) or 1 (for the genes downregulated by IL-6). In this way, we ensured that the transformed data points were spread throughout the entire interval [0,1]. We also conducted model calibrations with modified values of *m* for the genes downregulated by IL-6. Increasing *m* led to worse fitting results (MSE of approximately 0.025 for *m* = 0.7), while decreasing *m* produced fitting results with similar MSE but unsatisfying spread of the data over the interval [0,1].

### Statistical methods

Statistical significance of (phospho)proteomic and gene expression changes was analyzed by grouped t-test (two-tailed). Spearman correlation coefficients (r_s_) were calculated for averaged fold changes from three independent experiments. Statistical significance was defined as P<0.05. All statistical calculations were performed using GraphPad Prism software (version 5.04; GraphPad Software Inc., San Diego, CA).

## Supporting Information

S1 Fig Prior knowledge network comprising IL-6 signaling and downstream gene regulation. IL-6 signal transduction transitions were taken from the model by Ryll et al. [22]. This model was simplified by removing feedback loops as well as by deleting irrelevant input and output nodes (S1 Text). AND, OR, and NOT gates were transformed into simple activating or inhibitory transitions. We extended the model with signal transduction steps and gene-regulatory events based on newer literature (S3 Text). Color code: genes with measured expression level, black text/light-red ovals; proteins measured by RPA, white text/red ovals; proteins not measured, white text/grey ovals. The figure was created with Cytoscape [56].
(TIF)

S2 Fig Reduction curve for model family resulting from optimization of the PKN. The curve shows how the mean MSE (mean squared error) and the mean number of parameters of the model family depend on the selection threshold. The chosen setting of the selection threshold of 0.01 lead on average to approximately 110 model parameters. This figure was created with CNORfuzzy [34].
(TIF)

S3 Fig Heat map showing mean relative changes in gene expression (log2FC) upon IL-6 stimulation (IL-6 vs. control, 24 h) or siRNA-mediated knock down of RXRα (siRXRα vs. siControl, 72 h) in PHH of three independent donors (D9-D11; see Table 1 and Fig 6 for individual donor data). Asterisks indicate statistical significance: *, P<0.05; **, P<0.01; ***, P<0.005; ****, P<0.001.
(TIF)

S1 Table Predesigned TaqMan assays for quantitative gene expression analysis. (DOCX)

S2 Table Antibodies used for Western blot analysis. (DOCX)

S1 Text Summary of changes applied to the network by Ryll et al. (DOCX)

S2 Text Supplementary references used for compiling biological knowledge to the PKN. (DOCX)

S3 Text Detailed description of the CNORfuzzy method. (DOCX)
