# Supplementary figures and images for: Coordinating Role of RXRα in Downregulating Hepatic Detoxification during Inflammation Revealed by Fuzzy-Logic Modeling
Source: PLoS Comput Biol. 2016 Jan 4;12(1):e1004431. doi: 10.1371/journal.pcbi.1004431 (PMC4699813; doi:10.1371/journal.pcbi.1004431)

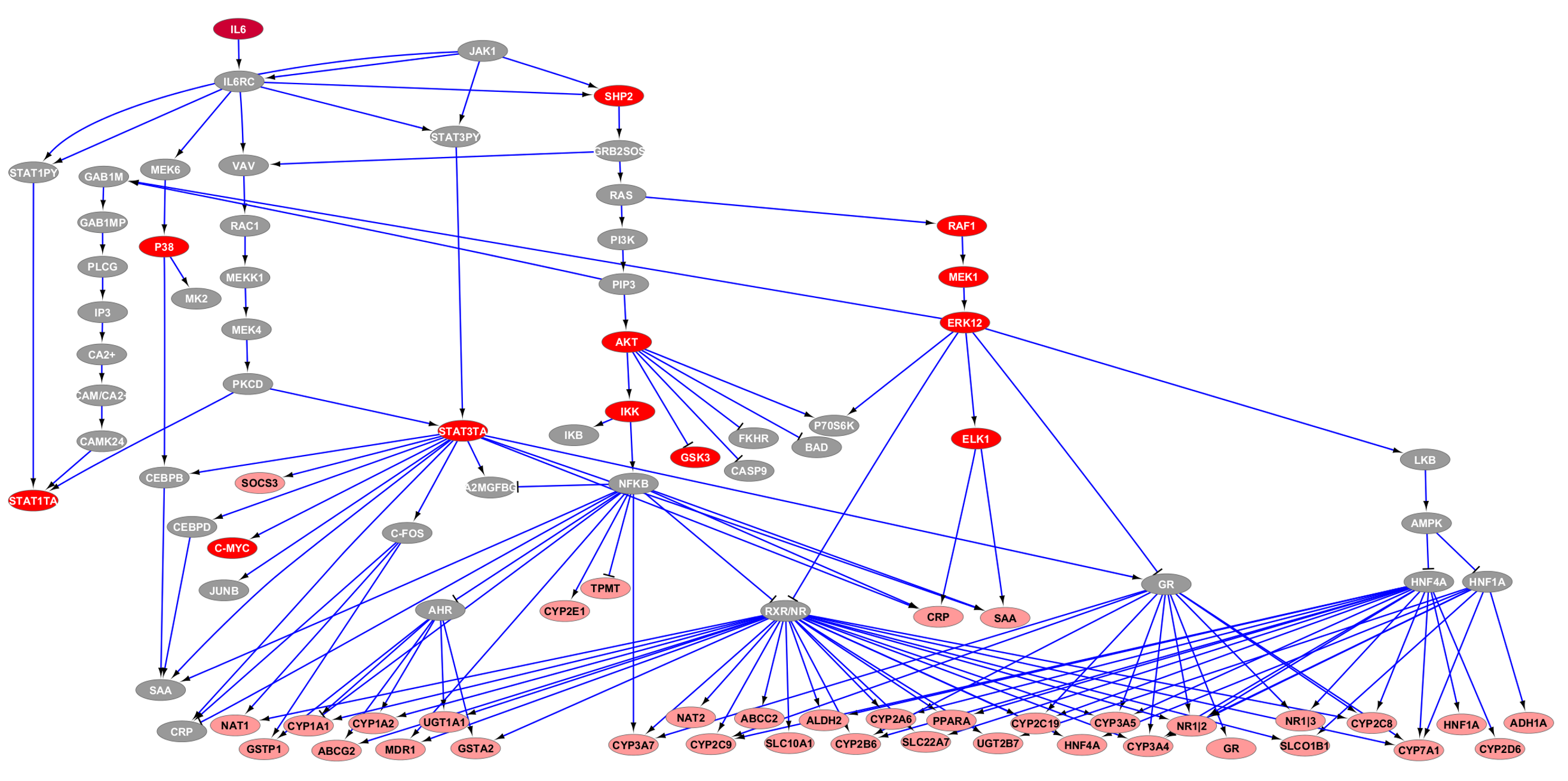

Supplement: S1 Fig — IL-6 signal transduction transitions were taken from the model by Ryll et al. [22]. This model was simplified by removing feedback loops as well as by deleting irrelevant input and output nodes (S1 Text). AND, OR, and NOT gates were transformed into simple activating or inhibitory transitions. We extended the model with signal transduction steps and gene-regulatory events based on newer literature (S3 Text). Color code: genes with measured expression level, black text/light-red ovals; proteins measured by RPA, white text/red ovals; proteins not measured, white text/grey ovals. The figure was created with Cytoscape [56]. (TIF) [file pcbi.1004431.s001.tif]

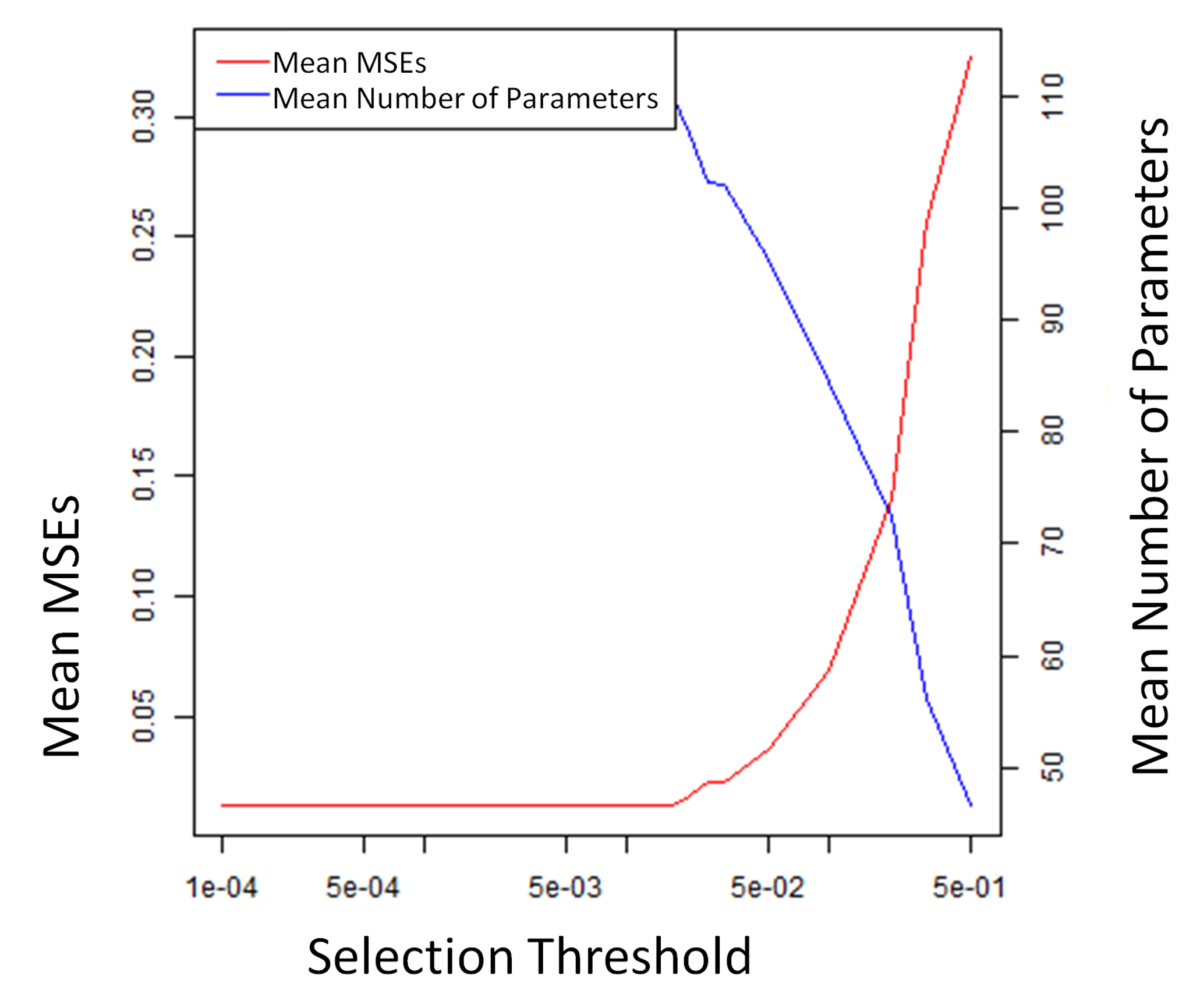

Supplement: S2 Fig — The curve shows how the mean MSE (mean squared error) and the mean number of parameters of the model family depend on the selection threshold. The chosen setting of the selection threshold of 0.01 lead on average to approximately 110 model parameters. This figure was created with CNORfuzzy [34]. (TIF) [file pcbi.1004431.s002.tif]

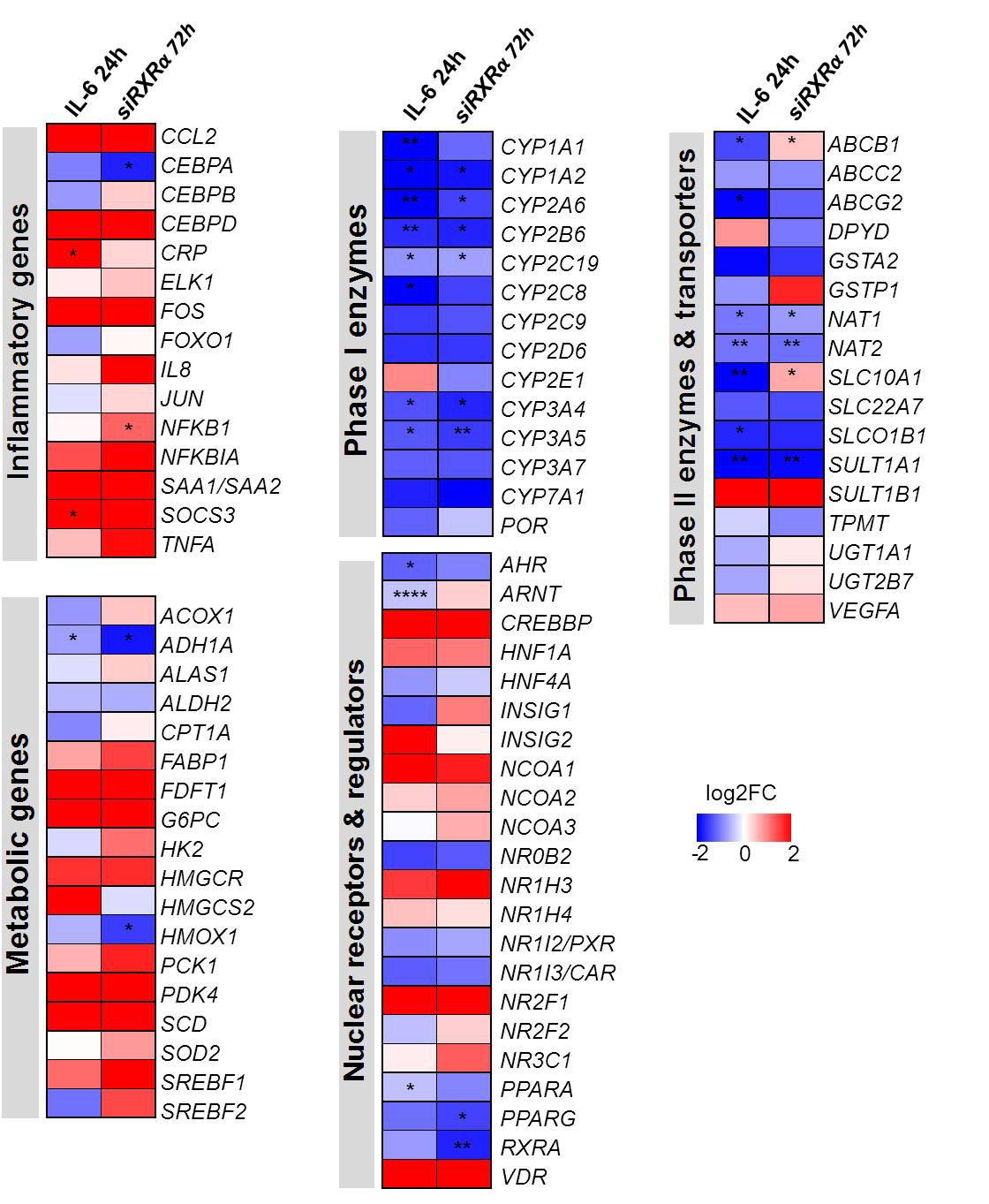

Supplement: S3 Fig — Asterisks indicate statistical significance: *, P<0.05; **, P<0.01; ***, P<0.005; ****, P<0.001. (TIF) [file pcbi.1004431.s003.tif]
