# Supplementary material for: Coordinating Role of RXRα in Downregulating Hepatic Detoxification during Inflammation Revealed by Fuzzy-Logic Modeling
Source: PLoS Comput Biol. 2016 Jan 4;12(1):e1004431. doi: 10.1371/journal.pcbi.1004431 (PMC4699813; doi:10.1371/journal.pcbi.1004431)
Supplement: S1 Table — (DOCX) [file pcbi.1004431.s004.docx]

**S1 Table:** Predesigned TaqMan^©^ assays used with the BioMark HD system (Fluidigm). All assays were purchased from Life Technologies, Carlsbad, CA, USA.

| **Gene symbol** | **Cat.-number** |
| --- | --- |
| *ABCB1* | Hs01067802_m1 |
| *ABCC2* | Hs00166123_m1 |
| *ABCG2* | Hs00184979_m1 |
| *ACOX1* | Hs01074241_m1 |
| *ADH1A* | Hs00605167_g1 |
| *AHR* | Hs00169233_m1 |
| *ALAS1* | Hs00167441_m1 |
| *ALDH2* | Hs00355914_m1 |
| *ARNT* | Hs01121918_m1 |
| *CCL2* | Hs00234140_m1 |
| *CEBPA* | Hs00269971_s1 |
| *CEBPB* | Hs00153133_m1 |
| *CEBPD* | Hs00270931_s1 |
| *CPT1A* | Hs00912671_m1 |
| *CREBBP* | Hs00231733_m1 |
| *CRP* | Hs00265044_m1 |
| *CYP1A1* | Hs00153120_m1 |
| *CYP1A2* | Hs01070374_m1 |
| *CYP2A6* | Hs00868409_s1 |
| *CYP2B6* | Hs03044634_m1 |
| *CYP2C19* | Hs00426380_m1 |
| *CYP2C8* | Hs00258314_m1 |
| *CYP2C9* | Hs00426397_m1 |
| *CYP2D6* | Hs00164385_m1 |
| *CYP2E1* | Hs00559367_m1 |
| *CYP3A4* | Hs00430021_m1 |
| *CYP3A5* | Hs01070905_m1 |
| *CYP3A7* | Hs00426361_m1 |
| *CYP7A1* | Hs00167982_m1 |
| *DPYD* | Hs00559279_m1 |
| *ELK1* | Hs00901847_m1 |
| *FABP1* | Hs00155026_m1 |
| *FDFT1* | Hs00926054_m1 |
| *FOS* | Hs00170630_m1 |
| *FOXO1* | Hs00231106_m1 |
| *G6PC* | Hs00609178_m1 |
| *GAPDH* | Hs99999905_m1 |
| *GSTA2* | Hs00747232_m1 |
| *GSTM1* | Hs01683722_gH |
| *GSTP1* | Hs00168310_m1 |
| *HK2* | Hs00606086_m1 |
| *HMGCR* | Hs00168352_m1 |
| *HMGCS2* | Hs00985427_m1 |
| *HMOX1* | Hs00157965_m1 |
| *HNF1A* | Hs00167041_m1 |
| *HNF4A* | Hs01023298_m1 |
| *INSIG1* | Hs01650977_g1 |
| *INSIG2* | Hs00379223_m1 |
| *JUN* | Hs00277190_s1 |
| *NAT1* | Hs00265080_s1 |
| *NAT2* | Hs00605099_m1 |
| *NCOA1* | Hs00186661_m1 |
| *NCOA2* | Hs06197990_m1 |
| *NCOA3* | Hs01105248_m1 |
| *NFKB1* | Hs00765730_m1 |
| *NFKBIA* | Hs00153284_m1 |
| *NR0B2* | Hs00222677_m1 |
| *NR1H3* | Hs00172885_m1 |
| *NR1H4* | Hs00231968_m1 |
| *NR1I2* | Hs00243666_m1 |
| *NR1I3* | Hs00901571_m1 |
| *NR2F1* | Hs00818842_m1 |
| *NR2F2* | Hs01047078_m1 |
| *NR3C1* | Hs00230818_m1 |
| *PCK1* | Hs00159918_m1 |
| *PDK4* | Hs01037712_m1 |
| *POR* | Hs00287016_m1 |
| *PPARA* | Hs00231882_m1 |
| *PPARG* | Hs01115513_m1 |
| *RXRA* | Hs00172565_m1 |
| *SAA1/SAA2* | Hs00761949_s1 |
| *SCD* | Hs01682761_m1 |
| *SLC10A1* | Hs00161820_m1 |
| *SLC22A7* | Hs00198527_m1 |
| *SLCO1B1* | Hs00272374_m1 |
| *SOCS3* | Hs02330328_s1 |
| *SOD2* | Hs00167309_m1 |
| *SREBF1* | Hs00231674_m1 |
| *SREBF2* | Hs00190237_m1 |
| *STAT3* | Hs00374280_m1 |
| *SULT1A1* | Hs00738644_m1 |
| *SULT1B1* | Hs00234899_m1 |
| *TNFA* | Hs00174128_m1 |
| *TPMT* | Hs00909011_m1 |
| *UGT1A1* | Hs02511055_s1 |
| *UGT2B7* | Hs00426591_m1 |
| *VDR* | Hs01045840_m1 |
| *VEGFA* | Hs00900055_m1 |
