# Supplementary material for: Coordinating Role of RXRα in Downregulating Hepatic Detoxification during Inflammation Revealed by Fuzzy-Logic Modeling
Source: PLoS Comput Biol. 2016 Jan 4;12(1):e1004431. doi: 10.1371/journal.pcbi.1004431 (PMC4699813; doi:10.1371/journal.pcbi.1004431)
Supplement: S2 Table — (DOCX) [file pcbi.1004431.s005.docx]

**S2 Table: Antibodies for Western blotting and their applied dilution**

| **Immunogen** | **Mol. weight** | **Host** | **Cat. #** | **Supplier** | **Dilution ^a^** | **Sec. ab.^b^** |
| --- | --- | --- | --- | --- | --- | --- |
| AKT pS473 | 60 kDa | rabbit | 9271 | CST | 1:1,000 | IRDye800 |
| ERK1/2-pT202/Y204 | 42/44 kDa | rabbit | 9101 | CST | 1:1,000 | IRDye800 |
| RXRα | 52 kDa | mouse | PP-K8508-00 | R&D | 1:1,000 | IRDye800 |
| STAT1pY701 | 84/91 kDa | rabbit | 9171 | CST | 1:1,000 | IRDye800 |
| STAT3pY705 | 79/86 kDa | rabbit | 9145 | CST | 1:2,000 | IRDye800 |
| β-Actin | 42 kDa | mouse | A5441 | Sigma-Aldrich | 1:5,000 | IRDye680/ 800 |

^a^ primary antibody, dilution in 1 % skim milk-TBST

^b^ secondary antibody, dilution: 1:10,000
