# Supplementary material for: Coordinating Role of RXRα in Downregulating Hepatic Detoxification during Inflammation Revealed by Fuzzy-Logic Modeling
Source: PLoS Comput Biol. 2016 Jan 4;12(1):e1004431. doi: 10.1371/journal.pcbi.1004431 (PMC4699813; doi:10.1371/journal.pcbi.1004431)
Supplement: S1 Text — (DOCX) [file pcbi.1004431.s006.docx]

# **S1 Text: Changes applied to the network by Ryll et al.**

Because our primary interest was in identifying system component interactions and not dynamic behavior, we simplified the network by removing feedback loops, which are by definition only relevant for dynamic network features. We also removed several input and output nodes of the network that were neither measured nor perturbed in the data used for model calibration. Therefore, these nodes would have been removed in any case by the compression step of the CNORfuzzy method. All these changes therefore did not affect the final modeling results.

- The following feedback loops were removed:
  - Feedback loops involving SOCS1 and SOCS3
  - Feedback loops from SHP2 to JAK/STAT, PI3K, MAPK pathways, and to IL6RC
  - Feedback loops from CAMK24 and MK2 to IL6RC
- Because inhibitors and activators were by default assumed to be inactive in the model by Ryll and colleagues [22], we did not measure them experimentally. The following input nodes (mainly activators and inhibitors) were therefore deleted:
  - GAB1_KIN
  - SIRP1A
  - GP130S
  - CYP_PTPE
  - PTEN
  - SHIP
  - PIAS1
  - PIAS3
  - SLIM
  - PHLPP
  - PDK1
  - ROS
  - MTOR, MTORC1, MTORC2
- Due to the removal of GP130S and the feedback loops from CAMK24 and MK2 to IL6RC the path from IL6 to IL6RC could be represented by a direct interaction edge.
- The following output nodes not connected to transcription factors in the network were also deleted:
  - IR
  - PRO_PROLIFERATIVE
  - PRO_HGF
  - ANTI_APOPTOTIC
  - IRS1_PY
  - IRS1_PS
  - VAR_APP
- In order to obtain an interaction network, which is the desired input for CNORfuzzy, all AND, OR, and NOT gates of the network were transformed into activating or inhibiting transitions from the input species of the gate to the output species.
