# Supplementary material for: Coordinating Role of RXRα in Downregulating Hepatic Detoxification during Inflammation Revealed by Fuzzy-Logic Modeling
Source: PLoS Comput Biol. 2016 Jan 4;12(1):e1004431. doi: 10.1371/journal.pcbi.1004431 (PMC4699813; doi:10.1371/journal.pcbi.1004431)
Supplement: S2 Text — (DOCX) [file pcbi.1004431.s007.docx]

**S2 Text: Supplementary Information references**

Agrawal A, Cha-Molstad H, Samols D, Kushner I (2001) Transactivation of C-reactive protein by IL-6 requires synergistic interaction of CCAAT/enhancer binding protein beta (C/EBP beta) and Rel p50. J Immunol Baltim Md 1950 166: 2378–2384.

Bolotin E (2010) HNF4α. Transcr Factor Encycl.

Cox AD, Der CJ. Ras family signaling: therapeutic targeting. Cancer Biol Ther. 2002;1: 599–606.

De Boussac H, Ratajewski M, Sachrajda I, Köblös G, Tordai A, et al. (2010) The ERK1/2-hepatocyte nuclear factor 4alpha axis regulates human ABCC6 gene expression in hepatocytes. J Biol Chem 285: 22800–22808. doi:10.1074/jbc.M110.105593.

Castellano E, Downward J (2011) RAS Interaction with PI3K: More Than Just Another Effector Pathway. Genes Cancer 2: 261–274. doi:10.1177/1947601911408079.

Chang F, Lee JT, Navolanic PM, Steelman LS, Shelton JG, et al. (2003) Involvement of PI3K//Akt pathway in cell cycle progression, apoptosis, and neoplastic transformation: a target for cancer chemotherapy. Leukemia 17: 590–603.

Delhase M, Li N, Karin M. Signalling pathways: Kinase regulation in inflammatory response. Nature. 2000;406: 367–368. doi:10.1038/35019154

Eulenfeld R, Dittrich A, Khouri C, Müller PJ, Mütze B, et al. (2012) Interleukin-6 signalling: more than Jaks and STATs. Eur J Cell Biol 91: 486–495. doi:10.1016/j.ejcb.2011.09.010.

Fessing MY, Krynetski EY, Zambetti GP, Evans WE (1998) Functional characterization of the human thiopurine S-methyltransferase (TPMT) gene promoter. Eur J Biochem FEBS 256: 510–517.

Ghose R, Zimmerman TL, Thevananther S, Karpen SJ (2004) Endotoxin leads to rapid subcellular re-localization of hepatic RXRalpha: A novel mechanism for reduced hepatic gene expression in inflammation. Nucl Recept 2: 4. doi:10.1186/1478-1336-2-4.

Hagihara K, Nishikawa T, Sugamata Y, Song J, Isobe T, et al. (2005) Essential role of STAT3 in cytokine-driven NF-kappaB-mediated serum amyloid A gene expression. Genes Cells Devoted Mol Cell Mech 10: 1051–1063. doi:10.1111/j.1365-2443.2005.00900.x.

Hayden MS, Ghosh S (2004) Signaling to NF-κB. Genes Dev 18: 2195–2224. doi:10.1101/gad.1228704.

Hennessy BT, Smith DL, Ram PT, Lu Y, Mills GB (2005) Exploiting the PI3K/AKT pathway for cancer drug discovery. Nat Rev Drug Discov 4: 988–1004. doi:10.1038/nrd1902.

Higgins LG, Hayes JD (2011) Mechanisms of induction of cytosolic and microsomal glutathione transferase (GST) genes by xenobiotics and pro-inflammatory agents. Drug Metab Rev 43: 92–137. doi:10.3109/03602532.2011.567391.

Hoesel B, Schmid JA (2013) The complexity of NF-κB signaling in inflammation and cancer. Mol Cancer 12: 86. doi:10.1186/1476-4598-12-86.

Hoque MT, Robillard KR, Bendayan R (2012) Regulation of breast cancer resistant protein by peroxisome proliferator-activated receptor α in human brain microvessel endothelial cells. Mol Pharmacol 81: 598–609. doi:10.1124/mol.111.076745.

Hösel M, Quasdorff M, Wiegmann K, Webb D, Zedler U, et al. (2009) Not interferon, but interleukin-6 controls early gene expression in hepatitis B virus infection. Hepatol Baltim Md 50: 1773–1782. doi:10.1002/hep.23226.

Israel A (2010) The IKK Complex, a Central Regulator of NF-?B Activation. Cold Spring Harb Perspect Biol 2. Available: http://www.ncbi.nlm.nih.gov/pmc/articles/PMC2829958/. Accessed 10 February 2014.

Jover R, Bort, Lechon, Castell (2002) Down-regulation of human CYP3A4 by the inflammatory signal interleukin-6: molecular mechanism and transcription factors involved.

Jover R, Moya M, Gómez-Lechón MJ (2009) Transcriptional regulation of cytochrome p450 genes by the nuclear receptor hepatocyte nuclear factor 4-alpha. Curr Drug Metab 10: 508–519.

Kast HR, Goodwin B, Tarr PT, Jones SA, Anisfeld AM, et al. (2002) Regulation of multidrug resistance-associated protein 2 (ABCC2) by the nuclear receptors pregnane X receptor, farnesoid X-activated receptor, and constitutive androstane receptor. J Biol Chem 277: 2908–2915. doi:10.1074/jbc.M109326200.

Lee HY, Suh YA, Robinson MJ, Clifford JL, Hong WK, et al. (2000) Stress pathway activation induces phosphorylation of retinoid X receptor. J Biol Chem 275: 32193–32199. doi:10.1074/jbc.M005490200.

Li D, Zimmerman TL, Thevananther S, Lee H-Y, Kurie JM, et al. (2002) Interleukin-1 beta-mediated suppression of RXR:RAR transactivation of the Ntcp promoter is JNK-dependent. J Biol Chem 277: 31416–31422. doi:10.1074/jbc.M204818200.

Migita K, Miyashita T, Maeda Y, Nakamura M, Yatsuhashi H, et al. (2005) An active metabolite of leflunomide, A77 1726, inhibits the production of serum amyloid A protein in human hepatocytes. Rheumatol Oxf Engl 44: 443–448. doi:10.1093/rheumatology/keh462.

Ozes ON, Mayo LD, Gustin JA, Pfeffer SR, Pfeffer LM, Donner DB. NF-kappaB activation by tumour necrosis factor requires the Akt serine-threonine kinase. Nature. 1999;401: 82–85. doi:10.1038/43466

Petrovic V, Teng S, Piquette-Miller M (2007) Regulation of drug transporters during infection and inflammation. Mol Interv 7: 99–111. doi:10.1124/mi.7.2.10.

Romashkova JA, Makarov SS. NF-kappaB is a target of AKT in anti-apoptotic PDGF signalling. Nature. 1999;401: 86–90. doi:10.1038/43474

Runge-Morris M, Kocarek TA (2009) Regulation of sulfotransferase and UDP-glucuronosyltransferase gene expression by the PPARs. PPAR Res 2009: 728941. doi:10.1155/2009/728941.

Ryll A, Samaga R, Schaper F, Alexopoulos LG, Klamt S (2011) Large-scale network models of IL-1 and IL-6 signalling and their hepatocellular specification. Mol Biosyst 7: 3253–3270. doi:10.1039/c1mb05261f.

Teng S, Piquette-Miller M (2005) The involvement of the pregnane X receptor in hepatic gene regulation during inflammation in mice. J Pharmacol Exp Ther 312: 841–848. doi:10.1124/jpet.104.076141.

Thomas M, Burk O, Klumpp B, Kandel BA, Damm G, et al. (2013) Direct transcriptional regulation of human hepatic cytochrome P450 3A4 (CYP3A4) by peroxisome proliferator-activated receptor alpha (PPARα). Mol Pharmacol 83: 709–718. doi:10.1124/mol.112.082503.

Tolson AH, Wang H (2010) Regulation of drug-metabolizing enzymes by xenobiotic receptors: PXR and CAR. Adv Drug Deliv Rev 62: 1238–1249. doi:10.1016/j.addr.2010.08.006.

TRANSFAC: Matys V, Kel-Margoulis OV, Fricke E, Liebich I, Land S, et al. (2006) TRANSFAC and its module TRANSCompel: transcriptional gene regulation in eukaryotes. Nucleic Acids Res 34: D108–D110. doi:10.1093/nar/gkj143.

Le Vee M, Jouan E, Stieger B, Fardel O (2013) Differential regulation of drug transporter expression by all-trans retinoic acid in hepatoma HepaRG cells and human hepatocytes. Eur J Pharm Sci Off J Eur Fed Pharm Sci 48: 767–774. doi:10.1016/j.ejps.2013.01.005.

Viatour P, Merville M-P, Bours V, Chariot A (2005) Phosphorylation of NF-kappaB and IkappaB proteins: implications in cancer and inflammation. Trends Biochem Sci 30: 43–52. doi:10.1016/j.tibs.2004.11.009.

Zanger, U.M., and Schwab, M. (2013). Cytochrome P450 enzymes in drug metabolism: Regulation of gene expression, enzyme activities, and impact of genetic variation. Pharmacol. Ther. *138*, 103–141.

Zhao, J., Liu, J., Pang, X., Wang, S., Wu, D., Zhang, X., and Feng, L. (2013). Angiotensin II induces C-reactive protein expression via AT1-ROS-MAPK-NF-κB signal pathway in hepatocytes. Cell. Physiol. Biochem. Int. J. Exp. Cell. Physiol. Biochem. Pharmacol. *32*, 569–580.

Zordoky, B.N.M., and El-Kadi, A.O.S. (2009). Role of NF-kappaB in the regulation of cytochrome P450 enzymes. Curr. Drug Metab. *10*, 164–178.
