# Supplementary material for: Coordinating Role of RXRα in Downregulating Hepatic Detoxification during Inflammation Revealed by Fuzzy-Logic Modeling
Source: PLoS Comput Biol. 2016 Jan 4;12(1):e1004431. doi: 10.1371/journal.pcbi.1004431 (PMC4699813; doi:10.1371/journal.pcbi.1004431)
Supplement: S3 Text — (DOCX) [file pcbi.1004431.s008.docx]

## S3 Text: Detailed description of the CNORfuzzy method

## We applied the CNORfuzzy method essentially as described by Morris et al. [1]. For convenience to the reader, we here summarize the most important steps of the applied fuzzy logic modeling algorithm.

## Simulation of a model in CNORfuzzy

The states of the species in a Boolean model are represented by discrete values (0 or 1), while in a CNORfuzzy model, states can be in a continuous interval [0,1]. Adapted Hill functions (also called transfer functions) can be applied to transform a Boolean to a CNORfuzzy model. The value *c* of a node *C* that depends only on node *A* having the value *a* is calculated as follows [1]:

$$c={(k}^{n}+ 1)\frac{a^{n}}{k^{n}+a^{n}}$$

In this function, *k* represents the midpoint of the function and *n* is the Hill coefficient. The following special cases can be considered:

If *C* is inhibited by *A*, the Hill function is subtracted from 1:

$$c={1-(k}^{n}+ 1)\frac{a^{n}}{k^{n}+a^{n}}$$

If *B* with value *b* is another input of *C* and *A* and *B* are OR connected, the value *c* is obtained by taking the maximum value of the Hill functions based on *A* (with index 1) and *B* (with index 2):

$$c=\max_{} \left( \left( {k_{1}}^{n_{1}}+ 1 \right)\frac{a^{n_{1}}}{{k_{1}}^{n_{1}}+a^{n_{1}}},\left( {k_{2}}^{n_{2}}+ 1 \right)\frac{b^{n_{2}}}{{k_{2}}^{n_{2}}+b^{n_{2}}} \right)$$

Correspondingly, in the case of an AND connection of *A* and *B*, the result of the logic gate is defined as the minimum of the two Hill functions:

$$c=\min_{} \left( \left( {k_{1}}^{n_{1}}+ 1 \right)\frac{a^{n_{1}}}{{k_{1}}^{n_{1}}+a^{n_{1}}},\left( {k_{2}}^{n_{2}}+ 1 \right)\frac{b^{n_{2}}}{{k_{2}}^{n_{2}}+b^{n_{2}}} \right)$$

If the input of those gate functions are only Boolean values (i.e., 0 or 1), the result is the same as in the Boolean logic.

## Network compression and expansion

The first two steps of the CNORfuzzy method involve network compression and expansion steps similar to the method in Cell Net Optimizer [2]. A given prior knowledge network (PKN) is compressed by trying to remove nodes that are neither experimentally measured nor perturbed in any experimental treatment and by fusing the respective interactions. Besides the measured and perturbed nodes, the compression method retains all nodes that are necessary for preserving logical consistency. This includes nodes with several incoming and outgoing interaction edges. The PKN at this point only contains interactions, but no logic gates. In the next step the PKN is transformed into a logical model. Inhibitory interactions are thereby represented by NOT gates. Several input nodes at the same node are by definition OR connected. Therefore, AND gates have to be added explicitly. In order to limit the number of added gates, only all possible AND connections consisting of two input nodes are added to the model.

## Model calibration

The following model calibration step consists of running a discrete genetic algorithm that determines transfer functions and a network topology in order to minimize the mean squared error (MSE) between the values simulated by the model and the normalized experimental data. The calibration method of CNORfuzzy not only involves the determination of the logic gates contained in the optimized model, but also the optimization of the respective Hill functions (i.e., the parameters *k* and *n*). As described by Morris et al. [1], the search space used in CNORfuzzy is restricted to seven different combinations of fixed values for *k* and *n* for each transfer function (n = 1.01, k = 68.5098 or n = 3 and k = 0.2, 0.3, 0.4, 0.5, 0.6, or 0.7), as these led to meaningful fitting results [1]. In addition to these seven transfer functions, the corresponding logic gate can also be inactive.

As stimulation of input species (IL-6 in our model) is usually assumed to be complete, these species are set at 1 by definition, leading to values of 1 in the Hill functions. This would cause all downstream gates to have values of 0 or 1, which is why linear transformation functions with estimated factors from [0,1] are used instead of Hill functions for input species. However, if the input species are 0, these transformation functions still produce values of 0 in the Hill functions leading to values of 0 or 1 for the computation of all downstream gates. Thus, if IL-6 is assumed inactive (i.e. control state) the simulated states will be 0 or 1 for all species. The factors of the transformation functions are estimated in the genetic algorithm together with the parameters of the Hill functions. The number of possible values for such a factor is also seven (0.2, 0.3, 0.4, 0.5, 0.6, 0.7, and 0.8) and again the corresponding gate can be inactive, as previously described [1].

Because the genetic algorithm can get stuck in a local minimum of the fitness function, and because several different models may yield a similar fitness, multiple runs of the genetic algorithm are necessary to determine the relevance of certain logic gates for fitting the model to the experimental data. A family of fuzzy logic models is the result of such multiple calibration runs, from which logical redundancies are finally removed.

## Model reduction and refinement

In the model reduction step, gates are removed from the optimized models, if this does not increase the MSE by more than a defined reduction threshold. This adaptation of the models decreases the number of model parameters. Several reduction thresholds are tested yielding a different model family for each reduction threshold.

Next, a refinement step is applied to find a local optimum for the model parameters near the "discrete" optimum obtained from the genetic algorithm. In CNORfuzzy the Subplex algorithm implemented in the R package *nloptr* [3] is used for that purpose. Finally, a selection threshold is chosen from the tested reduction thresholds such that the number of parameters is as low as possible without significantly increasing the mean MSE between model prediction and normalized experimental data.

## References

1. Morris MK, Saez-Rodriguez J, Clarke DC, Sorger PK, Lauffenburger DA. Training signaling pathway maps to biochemical data with constrained fuzzy logic: quantitative analysis of liver cell responses to inflammatory stimuli. PLoS Comput Biol. 2011;7: e1001099. doi:10.1371/journal.pcbi.1001099

2. Saez-Rodriguez J, Alexopoulos LG, Epperlein J, Samaga R, Lauffenburger DA, Klamt S, et al. Discrete logic modelling as a means to link protein signalling networks with functional analysis of mammalian signal transduction. Mol Syst Biol. 2009;5: 331. doi:10.1038/msb.2009.87

3. Johnson SG. The nlopt nonlinear-optimization package [Internet]. 2014. Available: http://ab-initio.mit.edu/nlopt
